# Biomarkers for Chimeric Antigen Receptor T Cell Therapy in Acute Lymphoblastic Leukemia: Prospects for Personalized Management and Prognostic Prediction

**DOI:** 10.3389/fimmu.2021.627764

**Published:** 2021-02-25

**Authors:** Ruimin Hong, Yongxian Hu, He Huang

**Affiliations:** ^1^ Bone Marrow Transplantation Center, The First Affiliated Hospital, Zhejiang University School of Medicine, Hangzhou, China; ^2^ Institute of Hematology, Zhejiang University, Hangzhou, China; ^3^ Zhejiang Province Engineering Laboratory for Stem Cell and Immunity Therapy, Hangzhou, China; ^4^ Zhejiang Laboratory for Systems and Precision Medicine, Zhejiang University Medical Center, Hangzhou, China

**Keywords:** chimeric antigen receptor T cell, relapse/refractory acute lymphoblastic leukemia, biomarkers, therapeutic response, adverse events

## Abstract

Chimeric antigen receptor (CAR) T cell therapy represents a breakthrough in immunotherapy with the potential of ushering in a new era in cancer treatment. Remarkable therapeutic response and complete remission of this innovative management have been observed in patients with relapse/refractory acute lymphoblastic leukemia. With CAR-T cell therapy becoming widely used both in multicenter clinical trials and as a commercial treatment, therapeutic efficacy monitoring and management of toxicities will be indispensable for ensuring safety and improving overall survival. Biomarkers can act not only as effective indicators reflecting patients’ baseline characteristics, CAR-T cell potency, and the immune microenvironment, but can also assess side effects during treatment. In this review, we will elaborate on a series of biomarkers associated with therapeutic response as well as treatment-related toxicities, and present their current condition and latent value with respect to the clinical utility. The combination of biomarker research and CAR-T cell therapy will contribute to establishing a safer and more powerful monitoring system and prolonging the event-free survival of patients.

## Introduction

Acute lymphoblastic leukemia (ALL) is a hematological malignancy that originates from clonal expansion of malignant B or T cell. The morbidity rates associated with ALL in the United States and China were reported as 1.7/100,000 and 0.69/100,000, respectively (1). Conventional treatments for patients with ALL include high-dose combined chemotherapy, targeted therapy, and allogeneic hematopoietic stem cell transplantation (allo-HSCT). Despite these standardized and intensive therapies, many patients still suffer from relapse, with the relapse rate counts for 15–20% in pediatric and 50% in adult B-ALL patients (2, 3). Refractory or relapse (RR) ALL remains the major cause of cancer-related mortality in children and adult patients. The 5-year survival rate of RR-ALL pediatric patients was 21 ± 0.8% and lower than 7% in adult patients (4). Allo-HSCT is the only therapy that offers the possibility of achieving long-term survival in these patients. However, nearly 90% of the RR-ALL patients missed the opportunity of allo-HSCT due to their poor tolerance to chemotherapy or failure to achieve complete remission before allo-HSCT.

**Figure 1:**
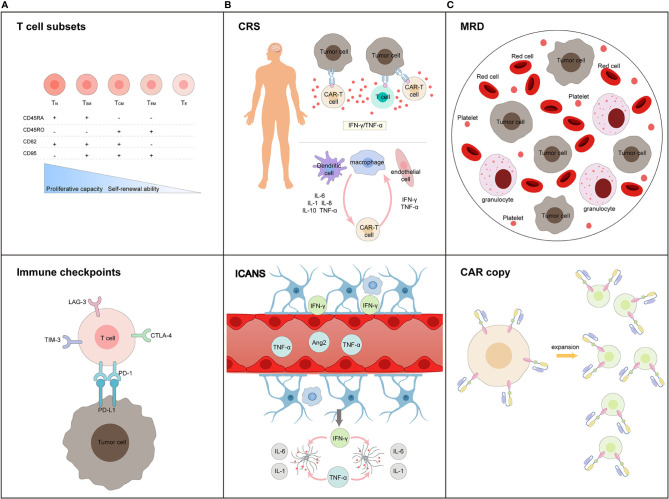
Biomarkers in terms of **(A)** efficiency prediction for CAR-T cells, **(B)** toxicity warning profile during treatment process, and **(C)** long-trem survival monitoring post-infusion. **(A)** T cell subsets and immune checkpoints expressed in T cells before CAR-T cell manufacturing are main factors influencing the efficiency of CAR-T cell products; **(B)** CRS and ICANS are two major adverse events during CAR-T cell therapy. The activation of T cells, CAR-T cells, dendritic cells, macrophages, and endothelial cells initiated these process. Inflammatory cytokines levels (including IFN-γ, TNF-α, IL-1, IL-6, IL-8, and IL-10) predicts the occurrence and severity of toxicity. **(C)** MRD monitoring, CAR-T cell expansion and persistence, are potential biomarkers of long-term survival.

In recent years, the increasing advancements and applications of cellular immunotherapy have enabled the use of chimeric antigen receptor (CAR) T cell therapy, and it has emerged as an efficacious method for the treatment of hematological malignancies (5, 6). CD19 CAR-T cell therapy for B-ALL achieved remarkable efficacy with a complete remission of 70–90% (7–9); multiple clinical trials using CD19 CAR-T therapy in RR-ALL have been summarized in **Table 1**. Nevertheless, clinical prognosis is heavily influenced by CAR-T cell function, tumor microenvironment, severe toxicities, primary resistance, and relapse. In addition, CAR-T cell features, including T cell subsets and stages of differentiation prior to engineering or in the final product, remain the major factors associated with CAR-T cell function (30). Therefore, early prediction, timely diagnosis, and effective intervention, together with the development of innovative CAR-T cell products, play a major role in managing these problems. In this review, we generalize biomarkers associated with therapeutic response and toxicities correlated with CAR-T cell therapy, which may be beneficial for evaluating the expansion and persistence of CAR-T cells, identifying adverse events, and predicting prognosis in RR-ALL patients (**Figure 1**).

**Table 1:** Clinical trials and outcomes of CD19 CAR-T cell therapy.

Institution	Author	No. pts	Diagnosis	Target	Costimulatory domain	Dose (cells/kg)	Lymphodepletion	Outcomes N (%)	NCT	Ref.	
MSKCC	Brentjens RJ	2	B-ALL	CD19	CD28	4×10^7^	Cy	CR: 1 (50%)	NCT01044069	(10)	
	Brentjens RJ	5	B-ALL	CD19	CD28	(1.5–3)×10^6^	Cy	MRD- CR: 5 (100%)				
	Davila ML	16	B-ALL	CD19	CD28	3×10^6^	Cy	CR: 10 (63%)		(11)	
	Park JH	53	B-ALL	CD19	CD28	N/A	FC/Cy	MRD- CR: 32 (67%) Grade 3–4 CRS/CRES: 14 (28%)/22 (42%)		(8)	
UPenn	Grupp SA	2	B-ALL	CD19	4-1BB	1.4×10^6^,1.2×10^7^	N/A	CR: 2 (100%) RR: 1 (50%)	NCT01626495	(12)	
	Maude SL	30	B-ALL	CD19	4-1BB	(0.76-20.6)×10^6^	N/A	CR: 27 (90%) Grade 3–4 CRS:8 (27%) CRES:13 (43%)	NCT01626495, 01622396	(13)	
	Teachey DT	51	B-ALL	CD19	4-1BB	1×10^7^, 5×10^8^	N/A	Grade 4–5 CRS: 30 (59%)	NCT02030847	(14)	
	Fitzgerald JC	39	B-ALL	CD19	4-1BB	N/A	N/A	Grade 3–4 CRS: 18 (46%)	NCT01626495	(15)	
	Maude SL	75	B-ALL	CD19	4-1BB	(0.2–5.4)×10^6^	FC	CR: 45 (60%)	NCT02435849	(7)	
	Gofshteyn JS	51	B-ALL	CD19	4-1BB	N/A	N/A	CRES: 23 (45%)	NCT01626495	(16)	
NCI	Lee DW	21	B-ALL NHL	CD19	CD28	1×10^6^, 3×10^6^	FC	CR: 13 (62%) Grade 3–4 CRS: 6 (29%) CRES:6 (29%)	NCT01593656	(9)	
	Fry TJ	21	B-ALL	CD19CD22	4-1BB	≥1 × 10^6^/Kg	FC	CR: 12 (57%) CRS: 16 (76%)	NCT02315612	(17)	
FHCRC	Turtle CJ	30	B-ALL	CD19	4-1BB	2×10^5^, 2×10^6^, 2×10^7^	FC, Cy, CE	MRD-CR: 27 (90%) Grade 3–5 CRS/CRES: 7 (23%)/15 (50%)	NCT01865617	(18)	
	Gust J	133	B-ALL NHL CLL	CD19	4-1BB	2×10^5^, 2×10^6^, 2×10^7^	FC	Grade 3–5 CRES: 28 (21%)	NCT01865617	(19)	
	Hay KA	53	ALL	CD19	4-1BB	2×10^5^, 2×10^6^	FC	CR:45 (85%) ≥Grade 3 CRS: 10 (19%)	NCT01865617	(20)	
	Hay KA	133	B-ALL NHL CLL	CD19	4-1BB	2×10^5^, 2×10^6^, 2×10^7^	FC	Grade 3–5 CRS: 16 (12%)	NCT01865617	(21)	
SCRI	Gardner RA	45	B-ALL	CD19	CD28	(0.5–10.0)×10^6^	Flu/Cy	MRD-CR: 43 (93%) Grade 3–4 CRS/CRES: 10 (22%)/9 (20%)	NCT02028455	(22)	
Chinese PLA General Hospital	Dai HR	9	B-ALL	CD19	4-1BB	N/A	N/A	MRD-CR: 3 (33%) Grade 3–4 CRS: 3 (33%) CRES:1 (11%)	NCT01864889	(23)	
	DAi HR	6	B-ALL	CD19/CD22	4-1BB	(1.7–3.0)×10^6^	FC	MRD-CR: 6 (100%)	NCT03185494	(24)	
Daopei Hospital China	Pan J	51	B-ALL	CD19	4-1BB	(0.05–14.0)×10^5^	FC	CR: 43 (84%)	ChiCTR-IIh-16008711	(25)	
The First Affiliated Hospital, Zhejiang University	Wei GQ	23	B-ALL	CD19	4-1BB	N/A	FC	MRD-CR: 20 (87%) ≥Grade 3 CRS/CRES: 9 (39%)/2 (9%)	ChiCTR-ORN-16008948	(26)	
The Affiliated Hospital of Xuzhou Medical University	Cao J	18	ALL	hCD19	4-1BB	1×10^6^	FC	MRD-CR: 12 (67%) Grade 3–5 CRS: 4 (22%) CRES: 1 (6%)	NCT02782351	(27)	
Southwest Hospital, Third Military Medical University	Heng G	10	ALL	hCD19	4-1BB	2.3×10^5^-4.17×10^7^	FC	CR:10 (100%) Grade 3–4 CRS: 4 (40%) CRES: 4 (40%)	NCT02349698	(28)	
Tongji Hospital, Huazhong University of Science and Technology	Wang N	89	B-ALL NHL	CD19 CD22 cocktail	CD28 and 4-1BB	CAR19: (2.6 ± 1.5)×10^6^ CAR22: (2.7 ± 1.2)×10^6^	FC	CR: 68 (76%) Grade 3–5 CRS: 19 (21%) Grade 3-5 CRES: 1 (1%)	ChiCTR-OPN-16008526	(29)	

Outcomes and incidence of adverse events of influential clinical trials in CD19 CAR-T cell therapy were shown in **Table 1**. ALL, acute lymphoblastic leukemia; Cy, cyclophosphamide; Flu, fludarabine; FC, cyclophosphamide and fludarabine; CR, complete remission; RR, relapse rate; CRS, cytokine release syndrome; CRES, CAR-related encephalopathy syndrome; MRD, minimal residual disease; MRD- CR, MRD-negative CR.

## Biomarkers for Therapeutic Response in CAR-T Cell Therapy

Except for patients’ overall survival (OS) and event free survival (EFS), the two main endpoints that efficacious for measuring treatment effects, there are growing interests in building feasible biomarkers in predicting short‐ and long‐term therapy outcomes. Various researches displayed that patients’ baseline characteristic, T cell function of CAR-T cell products and minimal residual disease (MRD) post CAR-T cell therapy were strongly associated with therapeutic response.

### Biomarkers for Patients’ Baseline Characteristics

Previous studies indicated that disease burden, high risk cytogenetic and molecular biology phenotype are major factors that may lead to poor response in RR-ALL patients after CAR-T cell therapy (8, 31). Lactate dehydrogenase (LDH), a key enzyme in the glycolytic pathway, is a negative prognostic biomarker in cancers (32). Increased serum LDH concentrations reflect a high tumor burden and proliferation in B cell malignancies and may be associated with aggressive disease dynamics (20). Besides, some evidence confirmed that elevated LDH levels may be related with an immunosuppressive tumor microenvironment, which could inhibit CAR-T cell function and result in tumor immune escape (27, 33). Hay and colleagues analyzed the factors correlated with durable EFS in adult B-ALL patients and suggested that a lower pre-lymphodepletion LDH and high platelet count were independent factors associated with better EFS. Their study also suggests that patients with higher pre-lymphodepletion LDH and lower platelet count may require systemic therapy before CAR-T cell infusion (20).

### Biomarkers for CAR-T Cell Function

CAR-T cell function are critical in maintaining an effective therapeutic response and durable remission. As reported, a differentiated T-cell phenotype, the expression of immune checkpoints (programmed cell death protein-1, PD-1; T cell immunoglobulin and mucin-domain containing-3, TIM-3; lymphocyte activation gene-3, LAG-3) as well as immune microenvironment can influence the anti-tumor activity, proliferation, and persistence of CAR-T cells (33, 34).

#### Biomarkers for T Cell Differentiation Level

T cell subsets can be divided into three main groups according to differentiation level, i.e., 1) stem cell memory T cells (Tscm), 2) central memory T cells (Tcm), and 3) effector memory T cells (Tem). These distinct subsets can be distinguished using polychromatic flow cytometry based on the presence of different surface markers (differentiation markers such as CD45RA, CD45RO, CD62L, CCR7, CD27, CD28, and activation markers such as CD25, CD127, CD57, and CD137) (35). Several studies have shown that less differentiated T cells exhibit stronger potential for expansion, persistence, and tumor eradication (18, 36, 37). Tscm retain greater stem cell-like function than any other memory T cell subset. For the same reason, CAR-T cells manufactured using less differentiated T cell subsets with functional characteristics of stemness and naivety, may exhibit improved expansion rates and extended persistence. Marianna S. et al. evaluated the function of CD19 CAR-modified CD8+ Tscm cells in a B-ALL mouse model and found that these CAR-T cell products mediated a prolonged antitumor response and increased survival compared to CD8^+^ T cells generated CD19 CAR-T cells (38). Xu et al. suggested that the expansion rate of CAR-T cells was positively correlated with the percentage of CD8+CD45RA+CCR7+ Tscm cells in CAR-T cell products (39). Furthermore, *in vivo* experiments indicated that the proportion of Tscm in the final CAR-T cell product was a positive marker for CAR-T cell expansion, whereas high frequency of Tem as well as CD57^+^ cells in the final product negatively impacted CAR-T cell expansion and anti-tumor activity (40).

#### Biomarkers for Immune Checkpoints

The assessment of the expression levels of PD-1, LAG-3, TIM-3, and their receptors indicated that high levels of these inhibitory molecules were associated with T cell exhaustion and poor response to CD19 CAR-T therapy (17). PD-1, a biomarker expressed on activated T cells, natural killer cells, and B cells, can inhibits T cell expansion, cytokine release, and cytotoxicity, thereby resulting in the immune escape of tumor cells (41–43). LAG-3 and TIM-3 are two next-generation immune checkpoint proteins expressed on different immune cell types and play a similar role in negatively regulating T cell activity (44, 45). Finney et al. compared T cell intrinsic factors between functional and dysfunctional responders and found that both group had similar frequencies of PD-1^+^ CD4^+^ CAR-T cells and PD-1^+^ CD8^+^ CAR-T cells, whereas the dysfunctional response group had a significantly higher percentage of LAG-3^+^ T cells and TIM-3^+^ T cells than the functional response group. In terms of apheresis products, higher frequencies of PD-1^+^LAG-3^+^ CD8^+^ T cells and PD-1^+^ CD4^+^ T cells were found in dysfunctional response group. Meanwhile, the results also indicated that high expression of LAG-3 combined with low secretion of TNF-α were associated with early therapeutic failure, and low frequency of TNF-α^+^/TIM-3^-^ CD8^+^ T cells in CD19 CAR-T cell products may be a risk factor for short persistence of CAR-T cells and early relapse (46). Fraietta and colleagues compared biochemical parameters in patients who achieved complete remission (CR), partial remission (PR), and non-response (NR) after CD19 CAR-T cell therapy. They demonstrated that patients with CR had significantly lower percentages of PD-1^+^ CD8^+^ CAR-T cells pre-infusion than those in PR and NR patients (37). This phenomenon was also confirmed in large B cell lymphoma or chronic lymphoblastic leukemia patients treated with anti-CD19 CAR-T cells (37, 47).

#### Biomarkers for Immune Microenvironment

Accordingly, a suppressive immune microenvironment may negatively influence the T cell function and correlate with a poor survival. Activation of both myeloid and lymphoid lineages may be an indicator of a less suppressed immune environment, which was favorable for the expansion and persistence of CAR-T cells. Enblad et al. treated fifteen B-ALL or B-cell lymphoma patients with CD19 CAR-T cells and found that patients with low monocytic myeloid-derived suppressor cell counts (CD14+CD33+HLA-DR cells) achieved better response. Moreover, patients exhibited higher levels of myeloid activation markers (IL-12, DC-Lamp) as well as lymphocyte effector markers (Fas ligand, TRAIL) had longer overall survival (48).

In addition, cytokines and chemokines secreted by polyfunctional T cells, including IFN-γ, MIP-1, IL-8, granzyme B, IL-17A, and IL-5, can mitigate immunosuppression caused by the tumor microenvironment and improve the clinical response in CD19 CAR-T cell therapy (49). Serum IL-15, MCP-1, and IL-7 levels can increase after conditioning chemotherapy, which is associated with CAR-T cell expansion potential *in vivo* and positive outcomes in patients treated with CD19 CAR-T cells (50). IL-12 is secreted by T cells, NK cells, dendritic cells, and macrophages. It increases the concentration of multiple inflammatory cytokines (such as IL-6, IL-8, IL-15, IL-18, IFN-γ, TNF-α, and GM-CSF) and enhances the cytotoxic functions of T cells and NK cells (51, 52). Kueberuwa et al. developed second-generation anti-murine CD19 IL-12-expressing CAR-T cells and introduced them into a mouse model with B cell malignancy. Nearly 25% of the mice achieved tumor eradication and long-term survival (53). IL-18—a cytokine similar to IL-12—mediates IFN-γ expression and regulates immune responses by activating monocytes and lymphocytes (54, 55). It can promote the antitumor activity of CAR-T cells by supporting the proliferation of CD8+ T cells (56) and reducing the number of immunosuppressive cells (57). Hu et al. developed an IL-18-expressing CD19 CAR-T cell product that exhibited enhanced proliferation and anti-tumor ability in a mouse model (56).

### Biomarkers for Long-Term Survival

CD19 CAR-T cell therapy has achieved remarkable therapeutic efficacy in RR B-ALL. Unfortunately, several individuals still failed to achieve CR or primarily resistance to CAR-T cell therapy, and relapse can occur in nearly 50% of B-ALL patients within 12 months after CAR-T cell infusion. Long-term survival of patients with ALL after CAR-T cell therapy is a primary outcome that reflects the overall prognosis and efficacy of CAR-T cell products. Previous studies indicated that CAR-T cell copy numbers, B cell aplasia (BCA), and MRD can serve as predictive biomarkers for long-term relapse-free survival after CAR-T cell infusion.

Transgene copies of CAR-DNA is an intuitional indication of CAR-T cell persistence and relate to the duration of therapeutic response after CAR-T cell infusion. Mueller et al. analyzed 79 patients with RR B-ALL infused with CD19 CAR-T cell products and evaluated CAR-T cell persistence based on CD19 CAR gene transgene copies. They demonstrated that patients who achieved CR experienced longer CAR-T cell persistence than non-response ones. The median duration of the two groups were 102 days and 27.8 days, respectively (58). Meanwhile, CD19 CAR-T cells can target all CD19-positive B cells and causes BCA of flexible duration. Hence, BCA usually act as a marker of *in vivo* CAR activity, is associated with prolonged remission after CAR-T cell treatment (59). Finney et al. treated 43 pediatric and adult RR-ALL patients with CD19 CAR-T cell therapy and demonstrated that the ongoing persistence of functional CD19 CAR-T cells or BCA for more than 6 months was a major determinant of durable remission, which was positively correlated with CD19 antigen burden at the time of infusion (46). Moreover, MRD monitoring is conventional and informative in B-ALL patients; MRD-negative CR after induction therapy, consolidation therapy, CAR-T cell therapy, and prior to allogeneic hematopoietic stem cell transplantation, significantly forebode better outcomes. Compared with flow cytometry, high-throughput sequencing (HTS) of IgH and TRG genes has higher sensitivity, which helped establish an optimized MRD threshold and identify patients with poor prognosis (58). Hay et al. studied 53 patients with RR B-ALL followed by CD19 CAR-T cell therapy and suggested that the absence of leukemia clone of IGH by HTS 3 weeks after CAR-T cell infusion in patients with MRD-negative CR is associated with improved EFS and OS (20).

Globally, these findings revealed that patients’ baseline disease status, T cell differentiation degrees, expression levels of PD-1, LAG-3 and TIM-3, immunological microenvironment combined with the CAR copy numbers and MRD monitoring were significant predictor factors associated with the clinical response to CAR-T cell therapy. Biomarkers related to short-term survival can potentially guide patient selection and optimization of CAR-T cell production before clinical application, additionally, biomarkers with long-term survival are essential for disease surveillance, directing immediate management and preventing relapse after CAR-T cell therapy.

## Biomarkers for Toxicities in CAR-T Cell Therapy

Cytokine release syndrome (CRS), immune effector cell-associated neurotoxicity syndrome (ICANS), coagulation disorder, secondary Hemophagocytic Lymphohistiocytosis (sHLH), hematologic toxicities, and infection are the side effects associated with CAR-T cell therapy, with CRS and ICANS being the most common. The incidence of CRS and ICANS in RR-ALL patients treated with tisagenlecleucel has been reported to be 77 and 40%, respectively (7, 60), with severe CRS and ICANS reached 47 and 15%, respectively. The median time of CRS onset was 2–3 days after CAR-T cell infusion (range: 1–22 days) (61, 62). High tumor burden, increased infusing dose, extent of lymphodepletion regimen, pre-existing endothelial damage, and resistant thrombocytopenia of B-ALL are important risk factors for the development and progression of CRS and ICANS (21, 63). The management of CRS and ICANS relies on its severity, which is mainly assessed using Common Terminology Criteria for Adverse Events (CTCAE) criteria and CAR-T cell therapy associated toxicity (CARTOX) criteria. Various studies have identified several biomarkers that can predict the development of adverse events after CAR-T cell therapy; thus, patients at risk can be closely monitored and receive timely prophylactic treatment (14, 19).

### Biomarkers for Cytokine Release Syndrome

CRS is a major complication of CAR-T cell therapy, and is characterized by systemic inflammation. CRS symptoms vary according to severity, ranging from mild disease with slight fever, fatigue, anorexia, nausea, vomiting, and headache, to severe disease with early onset high fever, hypotension, shock, disseminated intravascular coagulation, and even multiple organ dysfunction (60, 64). The management of CRS is mainly based on its severity grading, which is determined based on general symptoms, vital signs, and organ dysfunction. However, the variability of clinical symptoms and different self-perceptions among patients do not make them ideal candidates for precise CRS grading; therefore, specific biomarkers are needed for the monitoring and treatment of CRS.

CAR-T cells initially activate the effector cells and recipient immune system; CRS commonly develops in response to the binding of CAR-T cell receptors with specific antigens, which subsequently stimulate bystander immune cells and non-immune cells. Recent studies suggest that the crosstalk between the activation of the mononuclear/macrophage system and endothelial cells—which trigger an intense inflammatory cytokine storm—is primarily responsible for the development of CRS (11, 64, 65). Cytokine profiles related to CRS comprise not only effector cytokines including interferon (IFN)-γ, IL-2, IL-6, and granulocyte-macrophage colony stimulating factor (GM-CSF) but also of cytokines secreted by monocytes and/or macrophages —IL-1, IL-6, IL-8, IL-10, IL-12, tumor necrosis factor (TNF)-α, IFN-γ, monocyte chemotactic protein (MCP)-1, and macrophage inflammatory protein (MIP) 1α (66, 67). High levels of MCP-1 (≥1,343.5 pg/ml) with fever ≥ 38.9°C within 36 h of CAR-T administration are recognized as predictors of severe CRS and ICANS with the best sensitivity and specificity (21).

IL-6, IL-10, and IFN-γ are the strongest contributors to CRS development. IL-6 is a core cytokine in CRS pathophysiology, which enhances T cell proliferation and B cell differentiation as well as the production of ferritin and CRP (67, 68). IL-6 triggers the cytokine storm by binding to membrane-bound IL-6 receptors, which can form a complex with gp130 and initiate intracellular signaling in cells with/without membrane-bound IL-6 receptors (69, 70). IL-6 levels peaked when T cells reached maximal proliferation, and tocilizumab—an IL-6 receptor blocker—can relieve the symptoms of patients with life-threatening CRS (71–73). IFN-γ—secreted by activated T cells and tumor cells—plays a key role in mobilizing CRS after CAR-T cell infusion. The levels of IFN-γ and sgp130 increase early (3 days after infusion) in patients with severe CRS (14). IFN-γ also stimulates other immune cells, especially macrophages, which secrete proinflammatory cytokines, such as IL-6, IL-8, IL-12, IL-15, and TNF-α (74, 75), and the interaction between IFN-γ and macrophages aggravates CRS (64). In addition, recent studies have suggested that vascular endothelial activation is a risk factor associated with severe CRS. Hay et al. performed a study on 133 patients with RR CD19+ B cell malignancies who underwent CD19 CAR-T cell therapy, and demonstrated that serum VWF and Ang-2 concentrations were higher in patients with grade ≥4 CRS (21).

Except for these inflammatory cytokines, the levels of some serum biochemical parameters, such as C-reactive protein (CRP), ferritin, LDH, aminotransferase (AST), alanine aminotransferase (ALT), blood urea nitrogen (BUN), and creatinine, are elevated in patients with CRS and ICANS; however, these sometimes fail to predict the severity of the two toxicities (76–78). Teachey et al. demonstrated that patients with grade 4–5 CRS had higher peak levels of CRP and ferritin than those with grade 0–3 CRS, but CRP and ferritin did not improve CRS prediction in the first three days post CAR-T cell infusion (14). Davila et al. proposed CRP level ≥20 mg/dl as an indicator of severe CRS in the case of technological limitations with cytokine measurements (11). Our center also reported that the levels of CRP, serum ferritin, and D-dimer are associated with severe CRS, and their reduced levels indicate a promising response to tocilizumab or corticosteroids (79).

### Biomarkers for Immune Effector-Cell-Associated Neurotoxicity Syndrome

ICANS is another primary adverse event during CAR-T cell therapy, characterized by clinical manifestations, such as encephalopathy, aphasia, delirium, seizures, and tremor (80), which generally occur 1 to 28 days after CAR-T cell infusion. In some cases, ICANS can be concurrent with CRS. The incidence of ICANS was associated with high pretreatment disease burden, CAR-T cell expansion rate, and higher levels of pro-inflammatory cytokines. Ordinarily, mild to moderate ICANS is self-limited and can be controlled with close observation and supportive treatment. ICANS complicated with CRS usually presents a short duration and lower severity (41, 81). Severe ICANS can occur during the symptomatic improvement stage of CRS, with a high risk for acute cerebral edema, which may progress to delirium within a few hours and can even be fatal (19, 82, 83). Treatment strategies for ICANS include supportive care, or aggressive care with mechanical ventilation, high-dose corticosteroids, anti-epileptics, and medications for cerebral edema (84). The mechanism underlying the development of ICANS remains unclear, and it is now believed that the massive release of inflammatory cytokines and alterations in blood brain barrier permeability play a key role in the development of ICANS. Serum cytokine levels, including those of IL-6, IL-10, IFN-γ, TNF-α, and angiopoietin-2 (Ang-2) continuously increased in ICANS. Several studies have suggested that patients with severe ICANS may exhibit a higher concentration of IL-8, IL-10, and MCP-1 in their cerebrospinal fluid (80, 85, 86). High concentrations of numerous inflammatory cytokines can exert a direct effect on vascular endothelial cells, leading to increased epithelial permeability and dysfunction. Otherwise, CAR-T cells and inflammatory cells infiltration in the central nervous system together with activation of astrocytes and microglia may be the potential mechanisms responsible for ICANS (87).

Retrospective studies suggested that lymphodepletion regime, serum accumulation of cytokines (IL-6, MCP-1) within 24 h after CAR-T cell infusion combined with CD8+ T cell peak expansion predict the occurrence of severe ICANS (19, 88). High levels of IL-15 induced by intensive lymphodepletion, contribute to the maintenance of CD8+ memory T cell, which may enhance CAR-T cell proliferation as well as anti-tumor activity *in vivo* and result in advanced ICANS (19, 39). Santomasso et al. summarized data from 53 B-ALL patients after CD19 CAR-T cell therapy. They recommended using concentrations of IL-15, IL-10, and epidermal growth factor (EGF) in three days before CAR-T cell therapy to stratify patients into groups with different risk of severe ICANS. Patients with high levels of IL-15, IL-10, and low EGF comprising the high-risk group (80). These results reflected that severe ICANS may be correlated with more functional T cells, in terms of cytokine release after CAR-T cells stimulated by a large amount of tumor cells. Recent studies have shown that preexisting endothelial activation and severe thrombocytopenia (platelet <5–6 × 10^9^) are associated with severe CRS and ICANS, and these two factors may be connected to each other. Hay et al. posited that endothelial activation tends to occur in patients with low platelet count, which supports the fact that angiopoietin (Ang)-1 secreted by platelets contributes to the stability of endothelial cells (21, 80, 85). Conversely, von Willebrand factor (VWF) and Ang-2—two biomarkers secreted by Weibel-Palade bodies—play key roles in initiating coagulation and capillary leak, respectively (19, 89–91). Moreover, high Ang2:Ang1 ratio and vWF concentrations were found in patients with grade ≥4 ICANS (80, 85, 92). Therefore, endothelial activation biomarkers, including vWF, Ang-2, and endothelial-stabilizing biomarkers, such as Ang-1, and should be monitored before and after CAR-T cell infusion to monitor the incidence of CRS and ICANS.

In addition to these serum biomarkers, cytokines in CSF may reflect the immunological and biochemical dysfunction associated with ICANS. The activation of macrophages, microglia, astrocytes, and endothelial cells induces systemic inflammation and production of large amounts of quinolinic acid (QA), which results in increased levels of MCP1, IP10, IL-6, IL-8, IFN-γ, and INFα2 (80, 93). In addition, a high density of IFN-γ results in human brain microvascular pericyte stress, IFN-γ combined with TNF-α stimulates the secretion of IL-6 and VEGF from pericytes, an event that further promotes endothelial activation (19).

### Biomarkers for Coagulation Disorder

Coagulation disorder is a less frequent side effect following CAR-T cell therapy, with disseminated intravascular coagulation (DIC) being the most severe and life-threatening, and requiring close monitoring, early diagnosis, and timely treatment (18, 94). Numbers of previous chemotherapy, high tumor burden, and a low baseline platelet count may be risk factors for coagulation disorders. Coagulopathy biomarkers including increased D-dimer, fibrinogen degradation products (FDP), decreased fibrinogen, prolonged prothrombin time (PT), activated partial thromboplastin time (APTT), and thrombin time (TT) were indicators of coagulation disorders. Ying et al. found in their recent study that the incidence and severity of coagulation disorders were positively correlated with CRS grade, and persistent CRS may delay the recovery of coagulation. They also concluded that elevated IL-6, CRP, and ferritin levels were associated with increased PT, APTT, PT-INR, and TT to some extent (95). Due to the limited research on coagulation disorders after CAR-T cell treatments, detailed clinical and mechanistic studies should be conducted to further understand and manage this toxicity, which will help to control non-relapse mortality.

### Biomarkers for Secondary Hemophagocytic Lymphohistiocytosis

sHLH is a life-threatening hyperinflammatory syndrome induced by hyperactivated macrophages and lymphocytes, the exaggerated release of proinflammatory cytokines, as well as lymphohistiocytic tissue infiltration (96). sHLH occurs in patients with severe infections, malignancy or autoimmune diseases, characterized by prolonged hyperpyrexia, hepatosplenomegaly, pancytopenia together with hemophagocytosis in liver, spleen, and lymphoid tissue (97, 98). During the processes of CAR-T cell treatment, sHLH may occur secondary to severe CRS, with a incidence rate of 1–3.5% (99). However, it is difficult to dissect these two syndrome because of their similar clinical presentations and overlapping diagnosis criteria. Thus, biomarkers specific in sHLH are needed for the identification of HLH in patients with CRS after CAR-T cell therapy.

Patients with sHLH following CAR-T cell therapy showed an overexpression of serum cytokines produced by the aberrant activated immune system, including IFN-γ, TNF-α, IL-1, IL-4, IL-6, IL-8, IL-10, and IL-18, among which, the level of IFN-γ and IL-6 can be extremely high (100). Neelapu et al. proposed a reasonable solution in diagnosing sHLH in patients with severe CRS during CAR-T cell therapy, they recommended the peak serum ferritin measurement of >10,000 µg/L as a necessary criteria, and patients complicated with any two of the follow findings can be made the diagnosis: 1) grade > 3 increase in serum transaminases or bilirubin; 2) grade >3 oliguria or grade > 3 increase in serum creatinine; 3) grade > 3 pulmonary edema or histological evidence of hemophagocytosis in bone marrow or organs (84, 99). In conclusion, the substantially increased IFN-γ, IL-6 and ferritin combined with serum transaminases, bilirubin, creatinine, or soluble CD25 can be predictable biomarkers for sHLH associated with CAR-T cell therapy, and anti-IL-6 or humanized anti-IFNγ mAb may be feasible management for this disease (101).

### Biomarkers for Hematologic Toxicities and Infection

Hematologic toxicity has been reported after CD19 CAR-T cell therapy, which attributed mostly to the lymphodepleting chemotherapy regimen or CRS. The occurrence of neutropenia, anemia, and thrombocytopenia counted for 94, 51, and 80%, respectively (102). Fried S. et al. declared that perturbations in stromal cell-derived factor (SDF)-1 levels may correlate with late neutropenia (102). SDF-1 is a chemokine essential for regulating hematopoietic stem cell migration and survival, B-cell development and neutrophil migration (103, 104). Dunleavy K. et al. hypothesized that SDF-1 concentrations may decrease during rapid B-cell expansion, which resulted in reduced neutrophil migrating from the bone marrow to peripheral blood (103). Besides, a recent study profiled several cytokines correlated with the recovery of hematologic toxicities. Jain T. et al. compared the cytokines’ concentration between patients with and without complete count recovery 1 month after CAR-T cell therapy, they found that the former group had significantly higher peak levels of macrophage-derived chemokine (MDC). In addition, the fibroblast growth factor-2 (FGF-2), transforming growth factor-α (TGF-α), vascular endothelial growth factor (VEGF) as well as chemokines [macrophage inflammatory protein-1a (MIP-1a), and MIP-1b] also showed increased concentration in complete count recovery patients (105). Summarily, some chemokines and cytokines play a role in adjusting the marrow microenvironment and hematopoiesis, which may contribute to the recovery of hematopoietic progenitor cells.

Infection after CAR-T cell therapy is usually caused by persistent pancytopenia, abnormal immunity, severe CRS, and prior cytotoxic therapy (106–108). Infection presents as fever and elevated inflammation, which mirrors CRS. Severe infection is a high-risk factor associated with non-relapse mortality (109). Therefore, it is critical to identify biomarkers that can diagnose severe infection with high sensitivity and specificity. As described above, IL-6 was effective in predicting sCRS. Hui et al. showed that 9 of 11 patients with grade 4–5 infection exhibited a second IL-6 peak after the first CAR-related IL-6 peak, so they recommended “double peaks of IL-6” as a specific sign of severe infection. In addition, IL-8, IFN-γ, and IL-1β are predictive markers that further support the diagnosis of severe infection (110). Moreover, Diorio C. et al. presented a feasible predictive model combined with IFN-γ and IL1β, so as to differentiate between the analogous clinical entities of sepsis and CRS, the results displayed that an obviously elevated IFN-γ (>83 pg/ml) or a mildly elevated IFN-γ (<83 pg/ml) in combination with a low IL1β(<8 pg/ml) heralded the presence of CRS. Conversely, patients with IFN-γ<83 pg/ml and IL1β<8 pg/ml may complicated with sepsis (111). Current researches on infection and hematologic toxicity after CAR-T cell therapy are limited, further studies interpreting the underlying mechanisms and pathobiology will better the management of potential associated toxicities.

## Conclusion

CAR-T cell therapy has attained encouraging achievements in patients with RR ALL. A better understanding of biomarkers corresponding with selecting suitable patients, manufacturing CAR-T cell products, monitoring severe side effects, and predicting therapeutic response will play a valuable role in personally optimizing CAR-T cell therapy. Identification of new biomarkers could help in improving the quality of CAR-T cell products and establish a thorough understanding of the mechanisms associated with cytotoxicity and treatment response. With the progress in immunotherapy and systems biology technologies, biomarkers identified using genomics, proteomics, metabolomics, and transcriptomics will permit not only further comprehension of tumor heterogeneity but also the discovery of the cytotoxicity pathway. In summary, a suitable combination of biomarkers in CAR-T cell therapy will contribute to treatment management, durable responses, and durable overall survival.

## Author Contributions

HH, YH, and RH designed the article structure. RH was responsible for writing the manuscript and designing the figure. All authors contributed to the article and approved the submitted version.

## Funding

This work was supported by grants from the National Natural Science Foundation of China (81230014, 81470341, 81520108002), the Key Project of Science and Technology Department of Zhejiang Province (2018C03016-2), and the Key Research and Development Program of Zhejiang Province (2019C03016).

## Conflict of Interest

The authors declare that the research was conducted in the absence of any commercial or financial relationships that could be construed as a potential conflict of interest.
